# The mitochondrial malate dehydrogenase 1 gene *GhmMDH1* is involved in plant and root growth under phosphorus deficiency conditions in cotton

**DOI:** 10.1038/srep10343

**Published:** 2015-07-16

**Authors:** Zhi-An Wang, Qing Li, Xiao-Yang Ge, Chun-Lin Yang, Xiao-Li Luo, An-Hong Zhang, Juan-Li Xiao, Ying-Chuan Tian, Gui-Xian Xia, Xiao-Ying Chen, Fu-Guang Li, Jia-He Wu

**Affiliations:** 1 The State Key Laboratory of Plant Genomics, Institute of Microbiology, Chinese Academy of Sciences, Beijing, 100101, China; 2 Institute of Cotton Research, Shanxi Agricultural Academy of Science, Yuncheng, 044000, China; 3 The State Key Laboratory of Cotton Biology, Institute of Cotton Research, Chinese Academy of Agricultural Sciences, Anyang, Henan, 455000, China

## Abstract

Cotton, an important commercial crop, is cultivated for its natural fibers, and requires an adequate supply of soil nutrients, including phosphorus, for its growth. Soil phosporus exists primarily in insoluble forms. We isolated a mitochondrial malate dehydrogenase (*MDH*) gene, designated as *GhmMDH1*, from *Gossypium hirsutum* L. to assess its effect in enhancing P availability and absorption. An enzyme kinetic assay showed that the recombinant GhmMDH1 possesses the capacity to catalyze the interconversion of oxaloacetate and malate. The malate contents in the roots, leaves and root exudates was significantly higher in *GhmMDH1-*overexpressing plants and lower in knockdown plants compared with the wild-type control. Knockdown of *GhmMDH1* gene resulted in increased respiration rate and reduced biomass whilst overexpression of *GhmMDH1* gave rise to decreased respiration rate and higher biomass in the transgenic plants. When cultured in medium containing only insoluble phosphorus, Al-phosphorus, Fe-phosphorus, or Ca-phosphorus, *GhmMDH1*-overexpressing plants produced significantly longer roots and had a higher biomass and P content than WT plants, however, knockdown plants showed the opposite results for these traits. Collectively, our results show that *GhmMDH1* is involved in plant and root growth under phosphorus deficiency conditions in cotton, owing to its functions in leaf respiration and P acquisition.

Plant malate dehydrogenase (l-malate-NAD-oxidoreductase, MDH, EC 1.1.1.37) has multiple isoforms with distinct kinetic properties and physiological functions, and it is present in the cytosol, mitochondria, chloroplasts, and peroxisomes^1^. MDH catalyzes the interconversion of malate and oxaloacetate (OAA) coupled to the reduction/oxidation of the NAD pool. Although the direction of the reaction *in vivo* depends on the substrate/product ratios and the NAD redox state, it can vary within a given tissue according to the prevailing physiological state. The different MDH isoforms play a role in the transport and utilization of malate and OAA and the availability of NAD in various organelles. Functional redundancy among the isoforms can result in cooperation across organelles through malate/OAA shuttling of reducing equivalents in various cellular metabolic pathways^2^.

Mitochondrial MDH (mMDH) is involved in two major processes in plants. As a standard tricarboxylic acid (TCA)-cycle enzyme, mMDH oxidizes malate from the fumarase reaction to OAA to ultimately form citrate. In the conversion of Gly to Ser, mMDH reduces OAA to malate and provides a supply of NAD^+^ for Gly decarboxylase^3^. These physiological functions and metabolic pathways of mMDH have been confirmed in biochemical studies using loss- and gain-of-function strategies. For instance, spontaneous null mutants of mMDH1 in soybeans (*Glycine max*) showed a yellow foliage phenotype associated with the removal of two of the three mMDH isoforms^4^. In tomatoes (*Solanum lycopersicum*), mMDH antisense transgenic plants had approximately 60% less mMDH in the mitochondria relative to the wild-type plants but this had positive effects on the photosynthetic activity, CO_2_ assimilation rate, and total plant dry matter in plants grown under long-day conditions^5^. However, the antisense transgenic plants grown under short-day conditions had stunted growth, possibly due to impaired photosynthesis^5^,^6^. Low dry root weights and respiration rates were reported for the antisense mMDH tomato plants by van der Merwe *et al.*^7^. Single- and double-knockout mutants of the mMDH isoforms in *Arabidopsis* showed that the *mmdh1*-*mmdh2* mutant had no detectable mMDH activity but was viable, although it was small and slow growing. Interestingly, this slow-growing *mmdh1*-*mmdh2* mutant had an elevated leaf respiration rate without loss of photosynthetic capacity, suggesting that the increased respiration in the leaves could partially account for the low net CO_2_ assimilation and slow growth rate of *mmdh1*-*mmdh2*^8^.

Many studies have proposed complex functions for MDH; for example, Tesfaye *et al.*^9^ reported that the overexpression of cytosolic MDH in alfalfa (*Medicago sativa*) plants led to a four-fold higher organic acid content in the roots and higher rates of organic acid exudation compared with WT, which could increase the aluminum (Al) tolerance via metal chelation in the soil. The overexpression of MDHs from *A. thaliana* (*amdh*) and *Escherichia coli* (*emdh*) in transgenic tobacco plants (*Nicotiana tabacum*) enhanced the level of malate synthesis and improved Al resistance^10^. In apples, the cytosolic *MDH* gene contributes to malate accumulation in transgenic apple calli and transgenic tomatoes by the regulating genes and enzymes associated with malate degradation and transport, gluconeogenesis, and the TCA cycle^11^. Research on MDHs suggests that changes in the quantities of the MDH isoforms can affect the metabolic flux of various organic acids and have broad effects on plant growth and development, including Al resistance and nutrient availability.

Phosphorus (P), which is mostly found in insoluble forms in soil, is one of the most important mineral macronutrients for various plant physiological processes^12^,^13^. The majority of P applied to soil as fertilizer is rapidly fixed into insoluble forms, rendering it unavailable to plants and promoting environmental pollution^14^,^15^. Therefore, improving plant P uptake from soil is important for increasing agricultural productivity and decreasing pollution. Plants have evolved various adaptations for accessing insoluble nutrients, including modifications in the root’s morphological characteristics^16^ and the production of exudates, such as phosphatase^17^,^18^ or organic acids^19^,^20^. Organic acids exuded into the rhizosphere can chelate metals, resulting in desorption of nutrients from the soil matrix, thus making it available for uptake by the roots in transgenic tobacco^21^,^22^ and transgenic alfalfa^9^.

In the present study, *GhmMDH1* was isolated and characterized using loss- and gain-of-function strategies. Our results showed that the slow growth observed in RNAi plants was related to increased respiration and decreased P acquisition. Compared with WT plants, *GhmMDH1-*overexpressing plants showed higher malate production and exudation, which increased the absorption of insoluble P. These data indicate that *GhmMDH1* plays a role in plant and root growth under phosphorus deficiency conditions. Meanwhile, our results suggest that *GhmMDH1* can be used as a candidate gene to breed cotton cultivars for increasing insoluble P absorption.

## Results

### Isolation and expression profile of *GhmMDH1* gene

To identify candidate genes involved in highly effective mineral absorption, a pool of genes that responds to low mineral nutrients was identified from *G. hirsutum* in our laboratory using a suppression subtractive hybridization (SSH)-PCR technique^23^. We obtained and sequenced approximately 320 clones, one of which was highly homologous to mMDH in other plants in the GenBank database and was designated as *GhmMDH1*. A full-length cDNA fragment was obtained using the 5′- and 3′- rapid amplification of cDNA ends (RACE) technique. *GhmMDH1* contains a 1130-bp fragment with an open reading frame (ORF) of 1014 bp, which putatively encodes a 328 amino acid protein with a predicted molecular weight of 35.50 kDa, an isoelectric point of 8.94, and an MDH-like domain. A mitochondrial target signal containing 27 amino acids was predicted in the N terminus of GhmMDH1 ( http://ihg.gsf.de/ihg/mitoprot.html). The *GhmMDH1* nucleotide and the deduced amino acid sequences are shown in Fig. S1. We searched for the mMDH1 isoforms in the AADD-genome sequence from *G. hirsutum* (private correspondence) and found four MDHs with high identities (Fig. S2).

We performed BLASTp searches to determine the similarities between the query sequence and those found in the NCBI nonredundant protein sequences database. Because plants contain large numbers of MDHs, we focused on homologies with BLAST scores greater than 582 (E value = 0). GhmMDH1 and nine homologs from nine species were employed to analyze the identities. An analysis of the phylogenetic tree showed that GhmMDH1 and the homolog from *Theobroma cacao* were classified into a group; and GhmMDH1 was more closely related to the MDHs of dicotyledonous plants than to those of monocots (Fig.S3a). An analysis of the amino acid alignments indicated that members of the MDH family from ten species shared a high homology (84-94%) with GhmMDH1 (Supplementary Fig. S3b).

**Figure 1:**
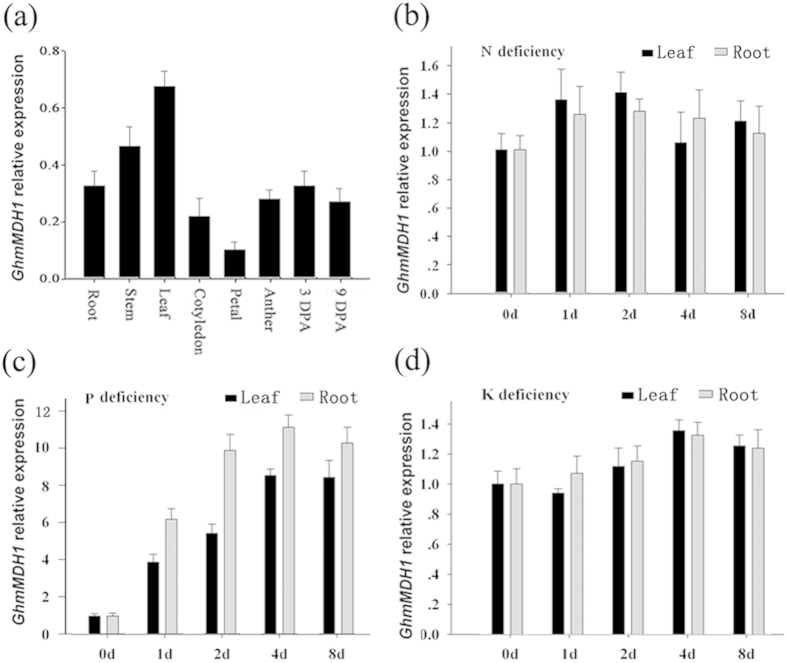
The expression profiles of*GhmMDH1.* (a) Various tissues from cotton; (b-d) N, P or K deficiency. DPA: days post anthesis. The values are the means ± standard deviation (SD) of three biological replicates.

Expression profiles of *GhmMDH1* in cotton were monitored using real-time RT-PCR. And *GhmMDH1* expression was observed in the roots, stems, leaves, cotyledons, petals, anthers, and developing fibers of 3-d post-anthesis (DPA) and 9-DPA in cotton plants. Preferential expression was exhibited in stems and leaves (Fig. 1a). To further assess the expression of *GhmMDH1* under nutrient deficient conditions, 20-d-old plants were transferred to Hoagland nutrient solution (HNS) free of nitrogen (N), P or potassium (K) to grow. The results showed that the expression levels of *GhmMDH1* were greatly induced in the leaves and roots 2 d after the P-deficient treatment, whereas under N or K deficiency, the expression levels did not differ (Fig. 1b–d).

### Subcellular localization of  GhmMDH1

**Figure 2:**
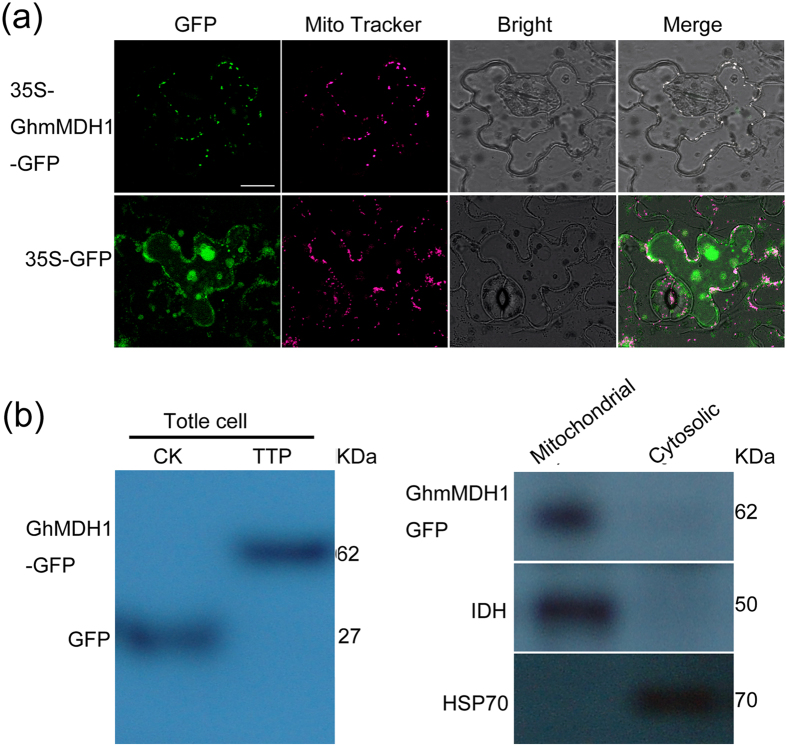
The subcellular localization of GhmMDH1 in the *N. benthamiana* leaves. (**a**) Confocal images of the subcellular localization of the GhmMDH1-GFP fusion protein through its transient expression in *N. benthamiana* leaves. MitoTracker Red dye was used to stain the mitochondria. Bars = 20 mm. (**b**) Immunoblots of the protein extract fractions from the transgenic *N. benthamiana* transiently expressing GhmMDH1-GFP. CK, leaves transiently expressing GFP; TTP, leaves transiently expressing GhmMDH1-GFP. IDH and HSP70 were used as the mitochondrial and cytosolic markers, respectively.

A mitochondrial target signal in the N terminus of the predicted GhmMDH1 protein contains a 27-amino acid sequence (1-MFRSVARSAAGKNLLRRGYATAVPERK-27) (Fig. S1). To examine the subcellular localization of GhmMDH1, we used a GFP-fused GhmMDH1 construct that was transiently expressed in *N. benthamiana* leaves. MitoTracker Red dye was used to stain the mitochondria, and confocal microscopy analyses revealed that the GFP-fused GhmMDH1 localized to the mitochondria in the *N. benthamiana* leaves (Fig. 2a). However, we detected the green fluorescence primarily in the cytoplasm of the *N. benthamiana* cells that transiently expressed the empty-vector control (GFP), which did not merge with the MitoTracker red fluorescence (Fig. 2a). Immunoblot analysis of the proteins extract fractions from the transiently transformed *N. benthamiana* leaves showed that the GFP and GhmMDH1-GFP were expressed in all of the cells (Fig. 2b). GhmMDH1-GFP was present in the mitochondrial fraction and but not in the cytosolic fraction (Fig. 2b). For positive controls, heat shock complex 70 and isocitrate dehydrogenase were detected in the cytosolic and mitochondrial fractions, respectively.

### *In vitro* GhmMDH1 activity assay

**Figure 3:**
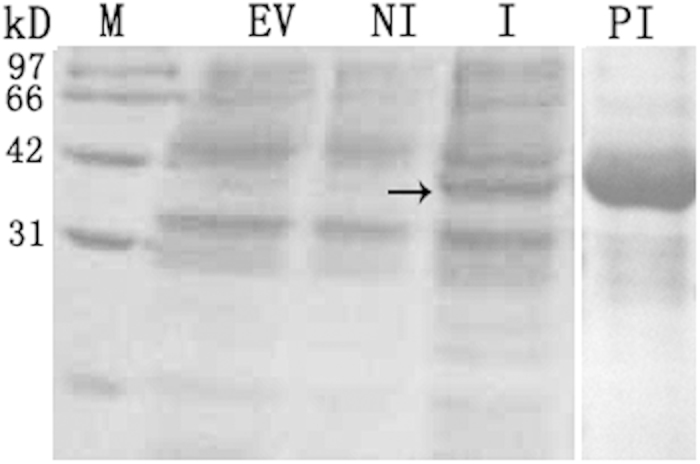
The recombinant GhmMDH1 following sodium dodecyl sulfate (SDS)-polyacrylamide (12%) gel electrophoresis. Lane M: the molecular weight marker; lanes EV, NI, I, and PI: the crude proteins extracted from *Escherichia coli* harboring the empty pET30a(+) control vector, non-inducible or inducible *E. coli* harboring the target vector, and the purified recombinant protein, respectively. The arrow points to the target protein.

To investigate the properties of GhmMDH1 *in vitro*, recombinant GhmMDH1 was obtained using *E. coli*-inducible expression, and the kinetic parameters of the purified protein were analyzed at pH 7.5 (Fig. 3, Table 1). The *V*_max_ values of OAA, NADH, malate, and NAD^+^ were similar, and the *K*_cat_ value of OAA was greater than the other substrates. The K_cat_/*K*_m_ of GhmMDH1 indicated that the specificity of OAA for the competing substrate was higher than malate. Together, these findings suggest that GhmMDH1 may drive the reaction toward the production of malate *in vitro*.

### Effects of *GhmMDH1* knockdown on plant growth and MDH activity

**Figure 4:**
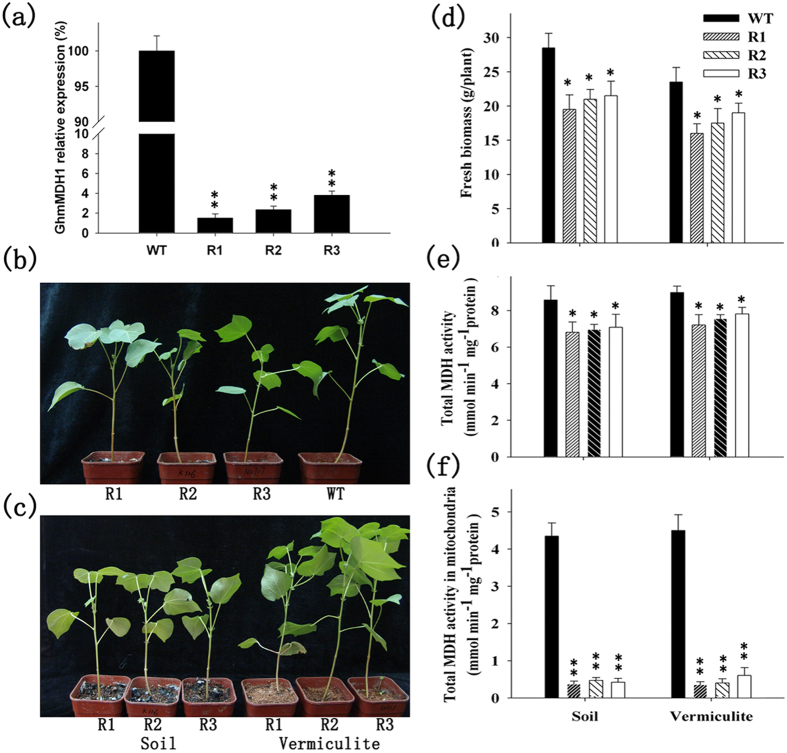
The growth inhibition and the reduction in the MDH activity in the RNAi transgenic lines relative to the wild type (WT). (**a**) The relative expression of *GhmMDH1* in the WT and RNAi lines; (**b**) the plant growth in soil; (**c**) the growth of the transgenic plants in soil or invermiculite soaked with Murashige–Skoog (MS) medium; (**d**) the biomass of the plants grown in soil or vermiculite; (**e**) the MDH activity in the plants grown in soil or vermiculite; (**f**) the MDH activity in the mitochondria isolated from the plants grown in soil or vermiculite. The values are the means ± SD of three biological replicates; the asterisks indicate significant differences compared with the WT (*P < 0.05, **P < 0.01).

To study the cellular role of GhmMDH1 in cotton, an RNAi experiment targeting *GhmMDH1* was performed using *Agrobacterium*-mediated transformation. We generated 25 independent transgenic plants using kanamycin resistance selection. Using PCR, qPCR, and genetic segregation analyses, three homozygous RNAi lines (R1, R2, and R3) were selected for the copy insertion (Table S1) and transcript silencing of *GhmMDH1* (Fig. 4a) and were used for further evaluation of the gene function. The growth of the plants in soil or vermiculite irrigated with Murashige–Skoog (MS) basal salt medium was marginally reduced in all of the RNAi lines compared with the WT (Fig. 4b). Interestingly, the size of the RNAi plants grown in soil was slightly smaller than those grown in the vermiculite supplied with MS medium (Fig. 4c). The biomasses of R1–R3 grown in soil or vermiculite + MS were 68–81% and 68–75% of the WT, respectively (Fig. 4d). The total MDH activity in the leaves of the RNAi lines was approximately 14–21% lower than that in the WT plants grown in soil or vermiculite (Fig. 4e). The MDH activity in the mitochondria isolated from the expanding leaves was also lower in all of the RNAi lines compared with the WT for both growth substrates (Fig. 4f).

### Effects of *GhmMDH1* knockdown on respiration rates

**Figure 5:**
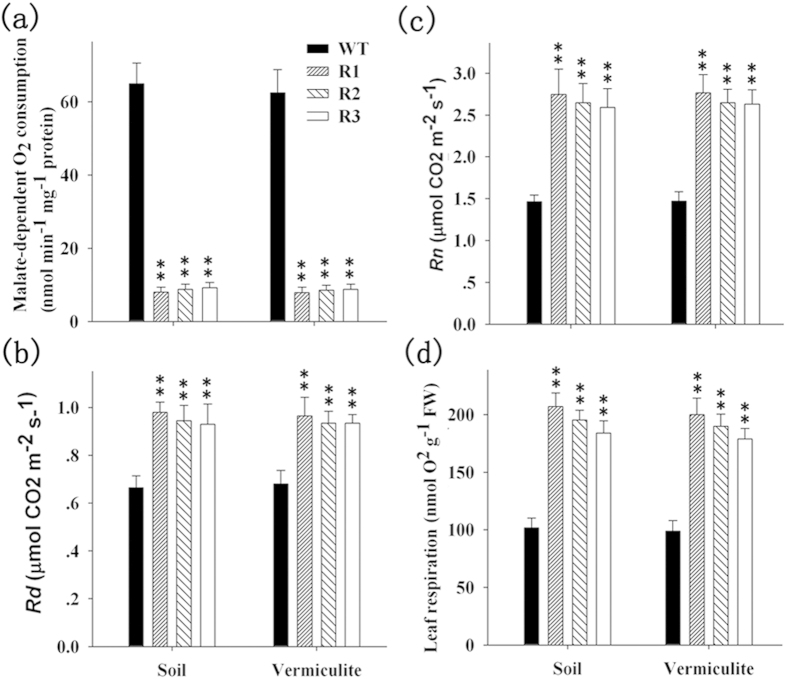
The effect of *GhmMDH1* knockdown on the respiration rates in the mitochondria and leaves in the WT and RNAi lines grown in soil or vermiculite. (**a**) The malate-dependent O_2_ consumption of the isolated intact mitochondria at pH 7.5; (**b**) the daytime respiratory CO_2_ production (*R*_*d*_) in the leaves; (**c**) the nighttime CO_2_ production (*R*_*n*_) in the leaves; (**d**) the leaf respiration as the oxygen consumption rate. The values are the means ± SD of three biological replicates; the asterisks indicate significant differences (*P* < 0.01) compared with the WT.

A few studies have shown that lower concentrations of mMDH correspond to changes in the plant growth that are due to alterations in the respiration rate and photosynthetic capacity^5^,^6^,^8^. Thus, we performed several experiments to analyze the respiration rate of transgenic cotton plants. Analysis of the malate-dependent oxygen consumption in isolated mitochondria revealed that the level of oxidation at pH 7.5 (at which the MDH activity is maximized and NAD-ME activity is minimized^8^) was 6.4 - to 7.5 - fold lower in R1–R3 than in the WT (Fig. 5a). However, the malate oxidation at pH 6.5 (at which NAD-ME is maximized and MDH is minimized^8^,^24^) was not significantly different (Fig. S4a).

Because of the effects of low GhmMDH1 on the rate of malate oxidation in the isolated mitochondria, we next assayed the effect of *GhmMDH1* knockdown on the total leaf respiration. The whole-leaf mitochondrial respiration was measured by the rate of CO_2_ release in the gas phase under the light^25^, and the respiration was 40–45% higher in R1–R3 than in the WT (Fig. 5b). In the dark, the rate of CO_2_ release was close to two-fold higher in the RNAi lines than in the WT (Fig. 5c). In addition, the respiration rates in the leaves of R1–R3, which measured as the oxygen consumption, were nearly double that of the WT leaves (Fig. 5d), whereas the root respiration rate did not differ between the RNAi lines and the WT (Fig. S4b). Collectively, these data suggest that the respiration rate is increased in RNAi plants in which *GhmMDH1* is knockdown.

To further characterize the RNAi lines, we analyzed the chlorophyll content and fluorescence parameter. Total chlorophyll content did not differ between the WT and RNAi lines in the soil or the vermiculite + MS (Fig. S4c). Similarly, the maximum potential quantum efficiency of Photosystem (PS) II, which was measured as the chlorophyll fluorescence parameters (*Fv/Fm*), did not differ significantly between the WT and RNAi lines (Fig. S4d). The results indicate that the photosynthetic apparatus is not apparently affected in the RNAi plants

Together, these findings suggest that the reduced plant growth in the RNAi lines can be partially attributed to the higher respiration rates. The biomass of the RNAi plants differed significantly between the soil and vermiculite medium (Fig. 4c,d) although the respiration rates were similar, indicating that other factors affected the growth of the RNAi lines. To explore this hypothesis, *GhmMDH1*-overexpressing transgenic lines were developed, the results of which are described below.

### The MDH enzyme activity and plant growth in *GhmMDH1*-overexpressing lines

**Figure 6:**
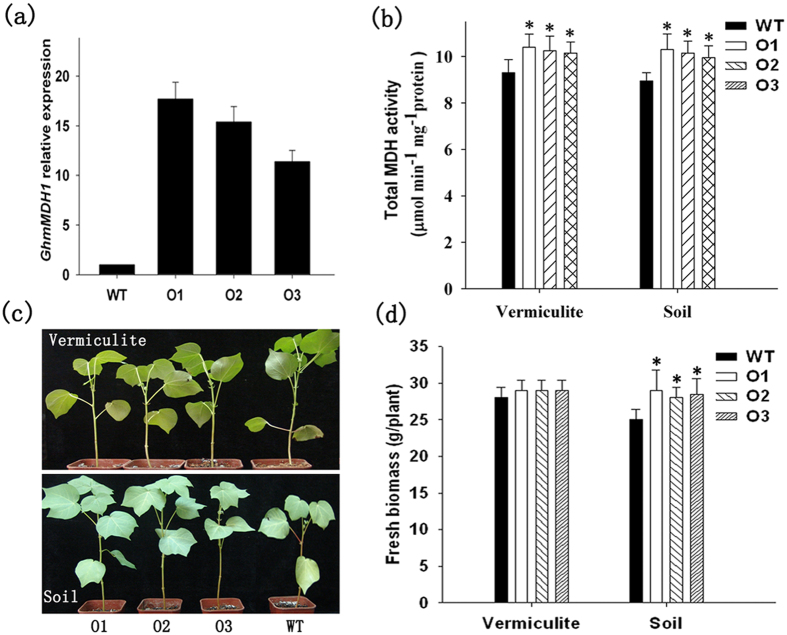
The growth and MDH activity of the *GhmMDH1*-overexpressing transgenic and WT plants grown in soil or vermiculite. (**a**) The relative expression of *GhmMDH1*; (**b**) the MDH activity; (**c**) the plant growth in vermiculite (upper panel) or soil (lower panel); (**d**) the plant biomass. The values are the means ± SD of three biological replicates; the asterisks indicate significant differences compared with the WT (*P < 0.05, **P < 0.01).

To further characterize the cellular function of *GhmMDH1*, the gene was overexpressed in cotton plants. The molecular analyses and selection of the homozygous lines for overexpression were performed as described previously^26^. Three lines (O1, O2, and O3) with a single-copy insertion and high expression of the target gene were selected using kanamycin resistance, PCR, and genetic segregation analyses (Fig. 6a, Table S1).

To confirm the effect of overexpression of *GhmMDH1* on the plant growth, we planted O1, O2, and O3 in pots with soil or vermiculite irrigated with MS medium. The total GhmMDH1 activity in the leaves of each line was 1.08 – 1.11 - fold higher than in the WT (Fig. 6b). Compared with the WT, the *GhmMDH1*-overexpressing plants did not differ in growth in the vermiculite, whereas, their growth was marginally increased in the soil, including significantly increase in the fresh biomass of the entire plant (Fig. 6c, d). Although the values of respiration rates according to analysis of oxygen consumption and CO_2_ release in O1-O3 lines are a little lower than those in WT, they did not significantly differ in the soil or vermiculite (Fig. S5a–d). The chlorophyll content and fluorescence parameters *Fv/Fm* in O1-O3 lines did not significantly differ with WT (Fig. S5e, f), indicating that the photosynthetic apparatus is not significantly affected in *GhmMDH1*- overexpressing plants.

### Analysis of malate production in transgenic cotton plants by HPLC

**Figure 7:**
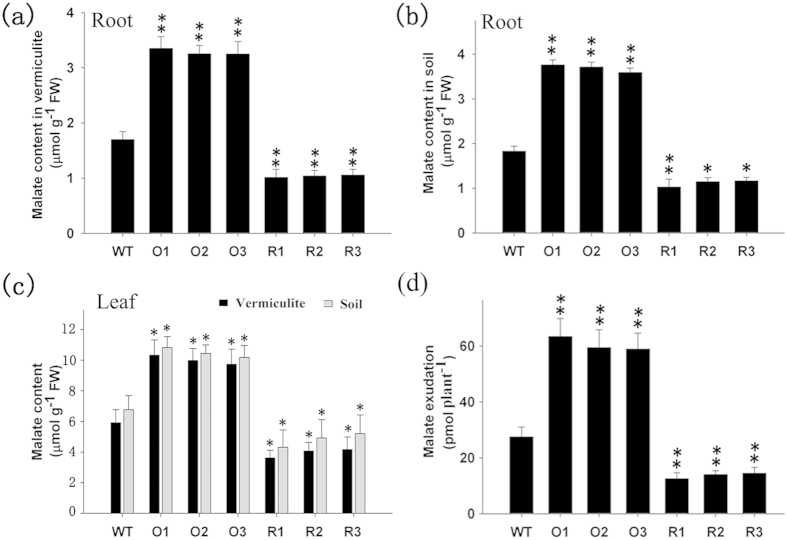
The malate contents in the roots, leaves and root exudates in the transgenic and WT plants grown in soil or vermiculite via HPLC. O1–O3: the *GhmMDH1*-overexpressing lines; R1–R3: the RNAi lines. (**a**) Roots, vermiculite; (**b**) roots, soil; (**c**) leaves, vermiculite and soil; (**d**) root exudates. The values are means ± SD of three biological replicates; the asterisks indicate significant differences compared with the WT (*P < 0.05, **P < 0.01).

We examined the malate content of the roots, leaves and exudates in the transgenic cotton plants using high performance liquid chromatography (HPLC). The malate content in the root tissue was significantly lower in the RNAi plants compared with the WT regardless of the growth medium, whereas that in the *GhmMDH1***-**overexpressing plants was significantly higher (Fig. 7a, b). In line with the results in the roots, the malate content in the leaf tissues of the transgenic plants also showed significant difference compared with the WT (Fig. 7c). The plants grown in soil had a higher malate content than the plants grown in vermiculite + MS, but these differences were not significant in either the roots or the leaves. The *GhmMDH1*-overexpressing plants exuded more malate than the WT plants grown in HNS, whereas the RNAi plants exuded less (Fig. 7d).

### Expression of *GhmMDH1* is correlated to the absorption of insoluble P

**Figure 8:**
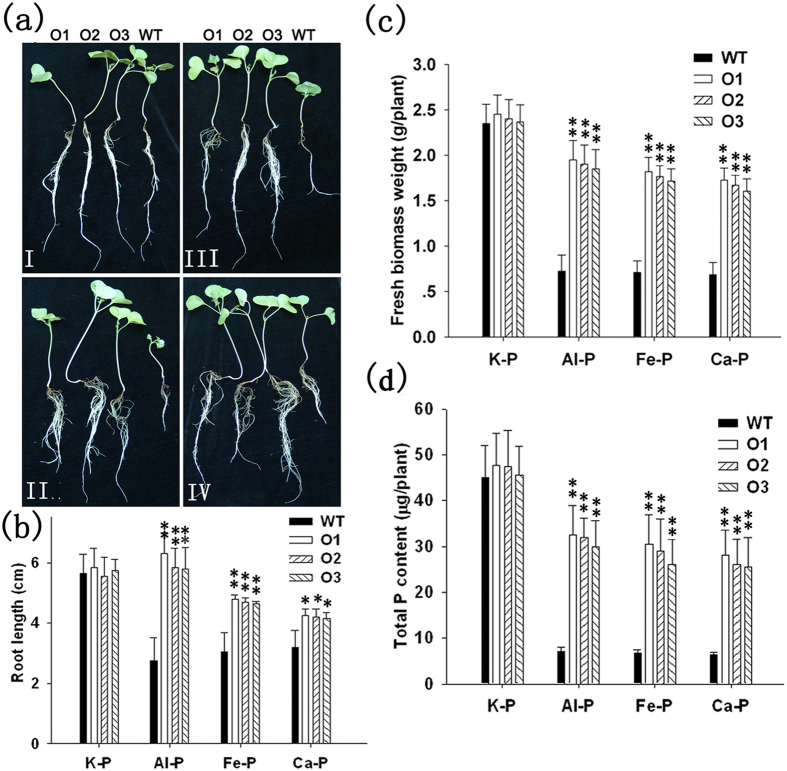
The effects of the different forms of insoluble P on the root length, plant biomass, and total P content in the WT and *GhmMDH1*-overexpressing transgenic cotton plants. (**a**) The growth patterns of the plants grown in 2 mM KH_2_PO_4_ (K-P, Panel I), Al-phosphate (Al-P, Panel II), Fe-phosphate (Fe-P, Panel III), or Ca-phosphate (Ca-P, Panel IV) medium. (**b**–**d**) The root length (**b**) fresh weight (**c**) and total P content (**d**) in the plants grown in the media described in (**a**). The values are the means ± SD of three biological replicates; the asterisks indicate significant differences compared with the WT (*P < 0.05, **P < 0.01).

**Figure 9:**
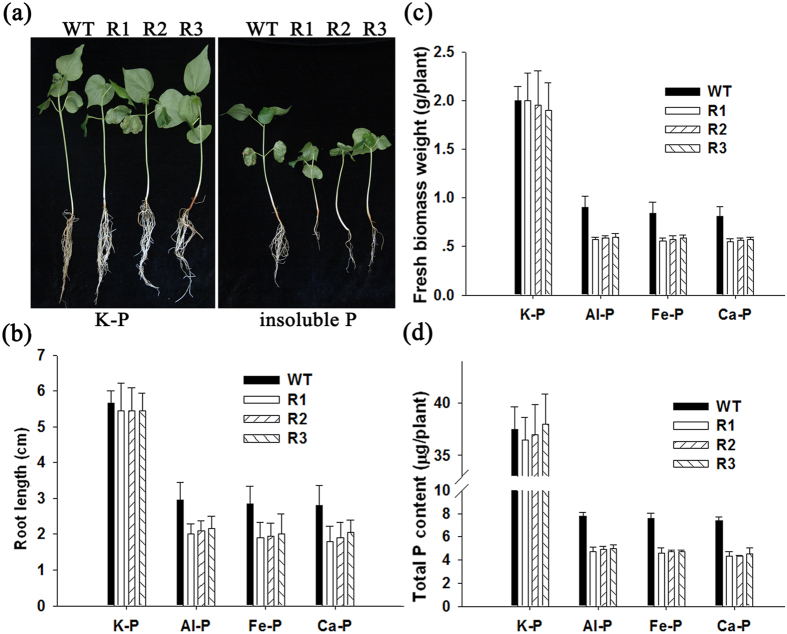
The effects of the different forms of insoluble P on the root length, plant biomass, and total P content in the WT and RNAi plants. (**a**) The growth patterns of the plants grown in soluble or insoluble P, as described in Fig. 8a(b–d). The root length (**b**), fresh weight (**c**) and total P content (**d**) in the plants grown in the media described in (**a**). The values are the means ± SD of three biological replicates; the asterisks indicate significant differences compared with the WT (*P < 0.05, **P < 0.01).

The malate that is released into the rhizosphere can chelate metals and promote P absorption from the soil by the roots^21^,^22^. The above results showed that the malate content in transgenic plants was positively correlated with the expression levels of *GhmMDH1* and GhmMDH1 activity. Therefore, we investigated the effects of the overexpression and knockdown of *GhmMDH1* on the ability of the transgenic plants to utilize insoluble P. The WT and transgenic plants had similar root growth, fresh biomass weight, and total P content when grown in HNS containing soluble P (2 mM KH_2_PO_4_) (Figs. 8 and 9). By contrast, when the plants were grown in HNS with insoluble P as Al-, Fe-,or Ca-phosphate, the root growth in the WT was reduced compared with the *GhmMDH1*-overexpressing plants (Fig. 8a-II–IV); the plants of three overexpressing lines had significantly longer roots compared with the WT (Fig. 8b). Similarly, the fresh biomass and P content of the *GhmMDH1*-overexpressing plants was significantly higher than the WT when grown with insoluble P (Fig. 8c, d). However, in the RNAi plants, the root length, biomass and P content were significantly lower than the WT when grown with insoluble P (Fig. 9). Taken together, the results demonstrate that the absorption of insoluble P in transgenic plants was positively correlated with the expression of *GhmMDH1*.

## Discussion

*In vitro*, recombinant GhmMDH1 protein favors the conversion of OAA to malate over the reverse reaction, as was observed in the significantly higher *K*_cat_/*Km* (specificity constant) values of OAA at pH 7.5. The kinetic properties of the recombinant MDH protein from other plants have shown similar results, including *Triticum aestivum*^27^,^28^, *M. sativa*^29^, *Ananas comosus*^30^, *Aptenia cordifolia*^31^, and *Malus domestica*^11^. Therefore, although the MDH reaction is reversible, *in vitro*, most recombinant MDH proteins drive the reaction toward the production of malate.

Although the reaction catalyzed by MDH favors malate production *in vitro*, it is unclear whether the direction of the reaction depends on the physiological conditions *in vivo*. Here, *GhmMDH1*-overexpressing plants showed higher MDH activity and malate levels, whereas RNAi plants showed lower MDH activity and malate levels compared with the WT. These results suggest that GhmMDH1 activity favors production of malate in cotton plant *in vivo*. Several other studies had reported that MDH-catalyzed reaction favored production of malate compared with OAA. For example, the overexpression of MDH increased malate accumulation in *Saccharomyces cerevisiae*^32^, and the overexpression of MDH1 from *Stylosanthes guianensis* increased malate concentrations in *S. cerevisiae*, and hairy roots in *G. max* and *Arabidopsis*^33^.

Our results suggest that GhmMDH1 may participate in the maintenance of respiratory rate by balancing the malate/OAA reaction in cotton. The knockdown of *GhmMDH1* in RNAi plants caused elevated respiration rates (Fig. 4b–d). However, this result is not at face value, which is consistent with the findings presented by others. Nunes-Nesi^5^ showed that lowering mMDH via antisense inhibition in tomatoes caused a decline in the respiration rates in the whole leaves. An analysis of the root respiration rates under the antisense inhibition of mMDH in tomatoes showed dramatic reductions in the respiration rate, which was in contrast to our findings in RNAi cotton plants^7^. These contradictory results may be due to different measurement and growth conditions. The direct CO_2_ gas-exchange measurements in mMDH tomato plants that have been silenced using antisense inhibition have not been reported. mMDH antisense transgenic plants show positive effects on the CO_2_ assimilation rate and total plant dry matter in plants grown under long-day conditions^5^; however, under short-day conditions, stunted growth and low dry root weight were reported^8^. Our data on cotton during a 12 photoperiod are more similar to those reported in short-day-grown versus long-day-grown tomatoes^5^,^7^, which is consistent with the results reported on mMDH mutants in *Arabidopsis*^8^.

Although the plant growth in the RNAi lines was inhibited, the level of inhibition varied between the plants grown in vermiculite with MS medium vs. the plants grown in soil. These results suggest that some soil nutrients could not be utilized by the RNAi plants. Previous studies have documented that the overexpression of genes related to organic acid synthesis^9^,^21^,^34^, phytase, and acid phosphatase^10^,^35^,^36^ could improve the P-acquising ability of transgenic plants. Our findings showing a positive relationship between the P acquisition with the expression of *GhmMDH1* and the malate content in the transgenic plants provide a case for the ability of plants to evolve various adaptations for accessing insoluble nutrients, which is similar to reports in alfalfa and tobacco^9^,^22^,^37^.

Most P that is applied to soil is immobilized in complexes with Al and Fe in acidic soils and with Ca in alkaline soil; therefore, only a small fraction is absorbed by the plants. In the present study, cotton plants overexpressing *GhmMDH1* grew better than the WT plants when supplemented with Al-phosphate, Fe-phosphate, or Ca-phosphate. The overexpression of *GhmMDH1* led to improved growth (more biomass) through enhanced root growth, which facilitated the P acquisition (Fig. 8). However, the RNAi plants showed negative effects in regards to the growth and P acquisition (Fig. 9). Root growth is important for the effective uptake of soil nutrients^10^,^38^. Because P is poorly mobile in soil, the ability of plants to acquire this nutrient largely relies on an effective root system to exploit the soil. Therefore, we propose that the superior growth of the transgenic cotton plants may be attributed in part to the greater exudation of malate, which leads to the more efficient chelation of Al, Fe, and Ca from their corresponding P compounds, and the subsequent desorption of P from these compounds. These results indicate that GhmMDH1-catalyzed excretion of malate could facilitate phosphorus acquisition in cotton, similar function has been reported for the citrate synthase. For example, the overexpression of citrate synthase in tobacco led to increased secretion of citrate and enhanced P acquisition from Ca-phosphate^21^. Koyama *et al.*^34^ documented that the overexpression of citrate synthase in *A. thaliana* had the same effect, with the P being absorbed from Al-phosphate.

## Methods

### Plant materials and treatments

Wild-type cotton (*G. hirsutum* ‘Zhongmiansuo 35’) (WT) and its transgenic lines (three RNAi and three overexpression lines) were used in this study. The seeds were sown in pots containing either vermiculite irrigated with MS medium or soil collected from a cotton field at the experimental farm of the Institute of Cotton Research, Shanxi Agricultural Academy of Science, Yuncheng, China (soluble P content of the soil = 5.2 mg/kg). The plants were grown at 28 °C, with a 12-h photoperiod and a light intensity of 150 μmol m^-2^ s^-1^ PPFD. The roots, stems, leaves, and cotyledons were collected from 2-wk-old seedlings, and the petals, anthers, and fibers were harvested at 3 and 9 DPA from greenhouse-grown plants. To assess the expression profile of *GhmMDH1* under nutrient deficient conditions, 20-d-old cotton seedlings were grown hydroponically in a plastic pot containing 30 ml of HNS respectively free of either N, P or K at 28 °C, with a 12-h photoperiod and a light intensity of 150 μmol m^-2^ s^-1^ PPFD. After 3 h of illumination, the leaves were collected from plants at 0, 1, 2, 4, and 8 d of growth under aseptic conditions. The collected materials were immediately frozen in liquid nitrogen and stored at –80 °C until use.

### Cloning of the *GhmMDH1* cDNA

The total RNA (2 μg) from young leaves was used in RACE experiments using 5′- and 3′-full RACE kits (TaKaRa, Dalian, China). The full-length cDNA of *GhmMDH1* was obtained using a 5′- and 3′- terminal RACE reaction according to the manufacturer’s protocol and based on the EST from the nutrient-stress EST pool. The coding sequence (CDS) of the gene was amplified according to the RACE sequence. The primers used for the gene isolation are shown in Table S2. The GhmMDH1 protein domain and motif analyses were performed using the National Center for Biotechnology Information BLAST database.

### Phylogenetic analysis

The protein sequences were aligned using Clustal X version 2.1^39^ with the default settings, and the neighbor-joining method was used to produce an unrooted phylogenetic tree in MEGA version 5.1^40^. A bootstrap test with 1000 replicates was used to evaluate the consistency of the analysis.

### RNA extraction and qRT-PCR

The total RNA from the cotton roots, leaves, stems, cotyledons, anthers, petals, and the 3- and 9-DPA fibers was extracted using ultracentrifugation^41^. For the cDNA synthesis, 2 μg of the total RNA from the different tissues was used for reverse transcription with Moloney murine leukemia virus reverse transcriptase according to the manufacturer’s recommendations (Promega, Madison, WI, USA). The qPCR assays were performed using SYBR Green Real-Time PCR Master Mix (Toyobo, Osaka, Japan) and a DNA Engine Opticon 2 Real-Time PCR Detection System (MJ Research). The cotton *GhUBI1* (GenBank accession no. EU604080) gene was used as an internal control. All of the reactions were performed in triplicate. The primers used in the qPCR are shown in Table S2.

### Subcellular localization via confocal microscopy

The coding sequence of GhmMDH1 was fused to GFP and cloned under the control of the 35S promoter in the plant expression vector pPZP111. The resulting pPZP111-GhmMDH1-GFP plasmid was transformed into *A. tumefaciens* strain GV3101 and infiltrated in *N. benthamiana* leaves. The GFP expression in the epidermal cells of the leaves was visualized using a confocal laser microscope (Leica SP8, Leica Microsystems). Mito Tracker Red FM (Invitrogen, CA, USA) was used for the mitochondrial staining, which was reported by Choi *et al.*^42^.

### Immunoblot analysis

The total, mitochondrial and cytosolic proteins were extracted from the leaf samples according to the methods described by Han *et al.*^43^ and Choi *et al.*^42^. After quantification using the Bradford assay (Bio-Rad protein assay kit), 15 μg of the total, mitochondrial or cytosolic proteins were subjected to SDS-PAGE. The immunoblot experiment was performed according to the methods described by Choi *et al.*^42^. Anti-GFP (Agrisera, Vännäs, Sweden) was used as the primary antibody, and isocitrate dehydrogenase and heat shock protein 70 antibodies (Agrisera) were used as the mitochondrial and cytosolic markers, respectively. The appropriate secondary antibodies conjugated with horseradish peroxidase (Sungene Biotechnology, Tian Jin, China) were used for the immunodetection.

### Expression of the recombinant GhmMDH1 and the enzyme kinetic property assay

The CDS of *GhmMDH1* that was amplified via PCR with the *CDS* special primer (Table S2) was inserted into the expression vector pET30a(+) at the *Bam*HI and *Sal*I sites. The recombinant GhmMDH1 protein was obtained using *E. coli*-inducible expression, separated using 12% sodium dodecyl sulfate-polyacrylamide gel electrophoresis, and purified using Ni-NTA His-Bind resin (Novagen, USA), according to the manufacturer’s instructions. The protein concentration was determined using Coomassie brilliant blue G-250, and the *K*_m_ and *V*_max_ values were determined using nonlinear regression^28^. The kinetic parameters for the OAA, NADH, malate, and NAD^+^ were determined according to Pastore *et al.*^28^ and Ding and Ma^27^. Briefly, for the reductive reaction, the reaction was performed at 25 °C in 2 mL of standard medium containing 0.2 mM NADH and the OAA concentration was increased, or the reaction was performed in standard medium containing 0.5 mM OAA and the NADH concentration was increased. For the oxidative reaction, 1.0 mM NAD^+^ was used and L-malate concentration was increased or 50 mM L-malate was used and the NAD^+^ concentration was increased. The reaction was monitored at 340 nm in 50 mM MOPS buffer (pH 7.5).

### MDH activity assay

Following 3 h of illumination, the young, fully expanded upper leaves of the transgenic and WT plants were collected to assay the total MDH activity. The leaf tissue was ground in liquid nitrogen and suspended in homogenization buffer (50 mM potassium phosphate [pH 7.0] containing 0.1 mM EDTA). After centrifugation at 3500 × *g* for 10 min in a microcentrifuge at 4 °C, the supernatants were used to determine the enzyme activity and protein content using the Bradford assay^44^. The total MDH activity was measured spectrophotometrically at 340 nm as described previously^29^. Briefly, the assay was performed using the following reaction buffer: 90 mM KH_2_PO_4_-KOH (pH 7.4), 0.05% (v/v) Triton X-100, 5 mM MgCl_2_, and 2 μM NADH. The reaction was initiated by the addition of 750 μM OAA. The cotton mitochondria were isolated from 3-wk-old seedlings according to Tomaz *et al.*^8^. The freshly isolated mitochondria (25 mg) were used for the determination of the latent mMDH activity in the same manner as described above.

### Plasmid construction and *Agrobacterium*-mediated cotton transformation

The CDS of *GhmMDH1* was inserted into the binary expression vector pBin438 under the control of the cauliflower mosaic virus (CaMV) 35S promoter to generate overexpression (35S:GhmMDH1) vectors. To construct the RNAi vector, a fragment of *GhmMDH1* was amplified using RNAi specific primers (Table S2) and cloned into the pHANNIBAL vector in the sense and antisense orientations to create a hairpin structure. The *Not*I-digested fragment was subcloned into the binary expression vector pART27. All of the vectors were introduced into *Agrobacterium tumefaciens* strain LBA4404.

*A. tumefaciens* LBA4404 harboring the different vectors was used to transform the explants of *G. hirsutum* ‘Zhongmiansuo 35’. The transformation of the cotton hypocotyl explants, the initiation of the calli, and the regeneration of the transformed plants were performed as previously described^45^ and the analysis of the transgenic plants was carried out as reported by Luo *et al.*^26^.

### Determination of the CO_2_ production and oxygen consumption

The rate of mitochondrial respiration in the light (day respiration, *R*_*d*_) was monitored according to Villar *et al.*^25^. Briefly, for each leaf, the photosynthetic rate was measured by decreasing the internal CO_2_ concentration (Ci) values (in the range 100-40 μL L^-1^) and at three different photo-flux densities (25, 45 and 70 μmol m^-2^ s^-1^ PPFD) at 25 °C and 60 to 70% humility. The linear regression of the photosynthetic rate vs Ci was calculated for each PPFD, and for each leaf, the intersected coordinate of the three linear regressions represent the *Rd* and the photosynthesis value. The rate of mitochondrial respiration in the dark (night respiration, *R*_*n*_) was determined according to Villar *et al.*^25^. Cotton leaves under normal condition were dark acclimated and measured for their CO_2_ evolution. In addition, the oxygen consumption by the detached leaves and roots and isolated mitochondria was measured using a Clark-type oxygen electrode for the respiration rates^8^.

### Analysis of the chlorophyll content and fluorescence parameter

The chlorophyll content and fluorescence parameter (*F*_V_/*F*_M_) were monitored to evaluate whether the photosynthetic apparatus are affected in the transgenic cotton leaves. The total leaf chlorophyll content was measured according to Tang *et al.*^46^, and the maximum potential quantum efficiency of Photosystem (PS) II of the leaves was measured as described previously^47^.

### Measurement of malate content and exudation by HPLC

To measure the malate content, the roots or leaves (1 g) were ground in liquid nitrogen and extracted with 10 ml of boiling 80% ethanol for 5 min. The homogenate was centrifuged at 13,000 × *g* at 4 °C for 10 min and the supernatant was filtered through a 0.45-μm filter. The malate content was analyzed via HPLC using an RP-18 column. The mobile phase was 0.01 M potassium dihydrogenphosphate and the flow rate was 0.8 ml·min^-1^ at 25 °C. the acids were detected by absorbance at 214 nm.

For the collection of the malate exuded from the roots, twenty 10-d-old cotton seedlings were grown hydroponically in a plastic pot containing 30 ml of HNS. Three pots were prepared for the WT and each transgenic line. After 15 d of growth under aseptic conditions, the culture medium was collected from each pot, concentrated, and analyzed using HPLC as described above.

### Effects of the different insoluble P sources on the growth and P uptake of the transgenic cotton

The seeds of the WT and transgenic lines were germinated and cultivated in 20 ml of sterile HNS with 2 mM Al-phosphate (pH 5.4), Fe-phosphate (pH 6.0), or Ca-phosphate (pH 7.8) as the sole P source. In addition, soluble P (2 mM KH_2_PO_4_) was added to the HNS as a control. After growing for 20 d under aseptic conditions, the plants were harvested to measure the root length and fresh biomass. The plant materials were oven dried at 65 °C for 2 h and then ashed at 180 °C for 16 h. The residues were then dissolved in 0.1 M HCl. The P concentration in the acid solution was measured using the molybdenum–antimony anti-spectrophotometric method^48^.

## Additional Information

**How to cite this article**: Wang, Z.-A. *et al.* The mitochondrial malate dehydrogenase 1 gene *GhmMDH1* is involved in plant and root growth under phosphorus deficiency conditions in cotton. *Sci. Rep.* **5**, 10343; doi: 10.1038/srep10343 (2015).

## Supplementary Material

Supplementary Information

## Figures and Tables

**Table 1:** The kinetic constants and parameters of the purified recombinant GhmMDH1 protein at pH 7.5.

**Substrate**	***V*_max_ (nmol min^−1^ mg^-1^)**	***K*_m_(mM)**	***K*_cat_ (min^−1^)**	***K*_cat_/*K*_m_ (mM^−1^ min^-1^)**
OAA	139 ± 4.1	0.132 ± 0.025	1215	9129
NADH	131 ± 3.8	0.107 ± 0.019	601	5616
Malate	161 ± 3.8	2.91 ± 0.29	928	319
NAD^+^	191 ± 4.1	0.766 ± 0.084	1015	1325

The values represent the mean ± standard deviation (SD) of three independent replicates
