# Supplementary material for: The mitochondrial malate dehydrogenase 1 gene GhmMDH1 is involved in plant and root growth under phosphorus deficiency conditions in cotton
Source: Sci Rep. 2015 Jul 16;5:10343. doi: 10.1038/srep10343 (PMC4503954; doi:10.1038/srep10343)

Supporting information

Additional Supporting information may be found in the online version of this article:

The mitochondrial malate dehydrogenase 1 gene *GhmMDH1* is involved in plant and root growth under phosphorus deficiency conditions in cotton.

Zhi-An Wang[1,2†], Qing Li[1†], Xiao-Yang Ge[3†], Chun-Lin Yang[1†], Xiao-Li Luo[2], An-Hong Zhang[2], Juan-Li Xiao[2], Ying-Chuan Tian[1], Gui-Xian Xia[1], Xiao-Ying Chen[1], Fu-Guang Li[3]*, Jia-He Wu[1]*

[1]The State Key Laboratory of Plant Genomics, Institute of Microbiology, Chinese Academy of Sciences, Beijing, 100101, China

[2]Institute of Cotton Research, Shanxi Agricultural Academy of Science, Yuncheng, 044000, China

[3] State Key Laboratory of Cotton Biology, Institute of Cotton Research, Chinese Academy of Agricultural Sciences, Anyang, Henan, 455000, China

†Co-first authors

*Correspondence:

Jiahe Wu, Tel/Fax: 86 10 64807375, E-mail: wujiahe@im.ac.cn;

Fuguang Li, E-mail: aylifug@163.com

Table S1 Genetic segregation of the six transgenic lines in T_2 derived from the original transgenic plants (T1)

Lines	Kan$^+$ plant no.*	Kan$^-$ plant no.	χ2 value	PCR$^+$ plant no.**	PCR$^-$ plant no.	χ2 value	Segregation rate
**R1**	106	36	0	42	13	0.0091	3:1
**R2**	125	43	0.0029	46	15	0.0082	3:1
**R3**	65	22	0.0057	39	12	0.0098	3:1
**O1**	145	51	0.023	43	15	0	3:1
**O2**	182	59	0.0047	47	17	0.0078	3:1
**O3**	121	39	0.0036	56	19	0.0008	3:1
**WT**	0	20	N/A	0	12	N/A	N/A

*, Kan$^+$ and Kan$^-$: kanamycin resistance and sensitivity. **, PCR$^+$ and PCR$^-$: PCR positive and negative.

Table S2. The primer sets used for isolation of target genes, PCR and qPCR

Gene names or treatment	Forward primer	Reverse primer
5' RACE	cgcggatcctccactagtgatttcactatagg	agatcgtcgcgagtcatac
3' RACE	cgcggatccacagcctactgatgatcagtcgatg	atcaagggtagttacacc
cDNA	gaaagaagccatcaaaccag	tttttttttggaaaaatagat
qPCR	cgaggatatgcaaccgccgt	gatgtggctgacatcagcgg
CDS	atgtttcgatccgttgctag	attctggttggcgaacttgat
RNAi	cacaccaaaagccaatttac	attctggttggcgaacttgat

GhUBI1	ctgaatcttcgctttcacgttatc	gggatgcaaatcttcgttaagac

Supplemental Figure legends

Figure S1. The nucleotide and deducing amino acid sequence *GhmMDH1* gene. The underlined amino acids represent mitochondrial signal peptide.

Figure S2. Identities and phylogenetic relationships between GhmMDH1 and its isoforms. (a) Amino acid sequences alignment of GhmMDH1 and its isoforms. The name and accession of GhmMDH1 and isoforms come from the gene ID numbers of AADD-genome of *G. hirsutum*. The letters in the same column with black background represent the same, those with red background represent 1 letter difference, and those with green background represent 2 letter difference. (b) The relatedness of GhmMDH1 and isoforms shown by a phylogenetic tree. The scale indicates branch lengths.

Figure S3. Phylogenetic relationships and identities between GhmMDH1 and MDHs in other plants (a) The relatedness of 10 mMDH homologs shown by an unrooted phylogenetic tree. The scale indicates branch lengths. (b) Amino acid sequences alignment of 10 MDH homologs. The name and accession of homologs are the same with (a). The letters in the same column with black background represent the same, those with red background represent less than 2 letter difference, and those with green background represent more than 3 letter difference.

Figure S4. Not impact of GhmMDH1 loss on roots respiratory rates and photosynthetic apparatus. (a) Malate-dependent respiratory rates of isolated intact mitochondria at pH 6.5. (b) Root respiration as oxygen consumption rate. (c) Total chlorophyll content in leaves. (d).

The Fv/Fm of leaves. Bars are means ±SD of two biology replicates.

Figure S5. Not effective of GhmMDH1 overexpression on respiratory rates and photosynthetic apparatus. (a) Respiratory CO2 production of lighted leaves. (b) CO2 production in darkened leaves. (c) Leaf respiration as oxygen consumption rate. (d) Root respiration as oxygen consumption rate. (e) Total chlorophyll content in leaves. (f) The Fv/Fm of leaves. Bars are means ±SD of two biology replicates.

Figure S1.

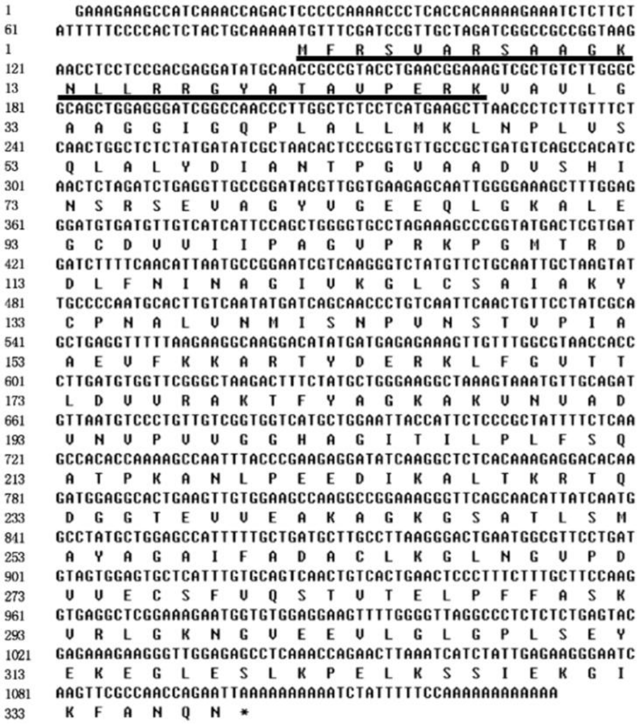

Figure S2

(a)

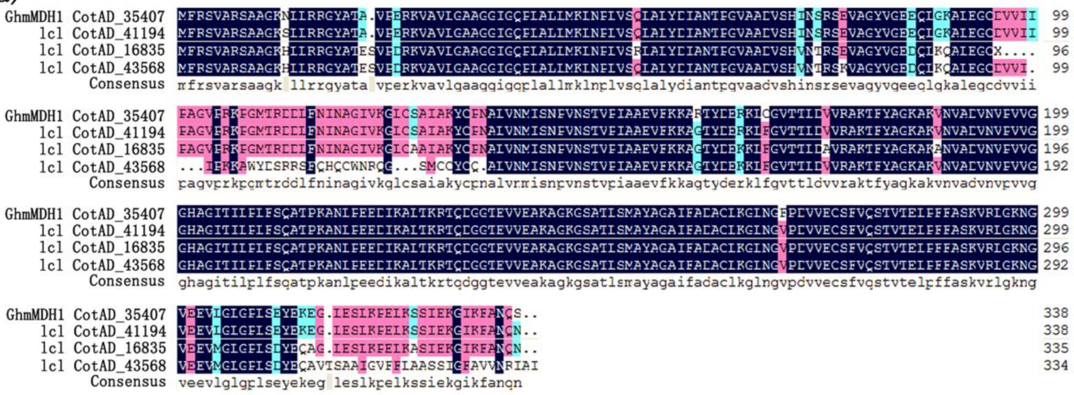

(b)

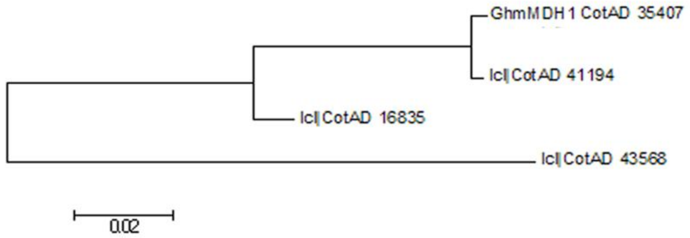

GhmMDH1 CotAD 35407
lcl|CotAD 41194
lcl|CotAD 16835
lcl|CotAD 43568
0.02

Figure S3.

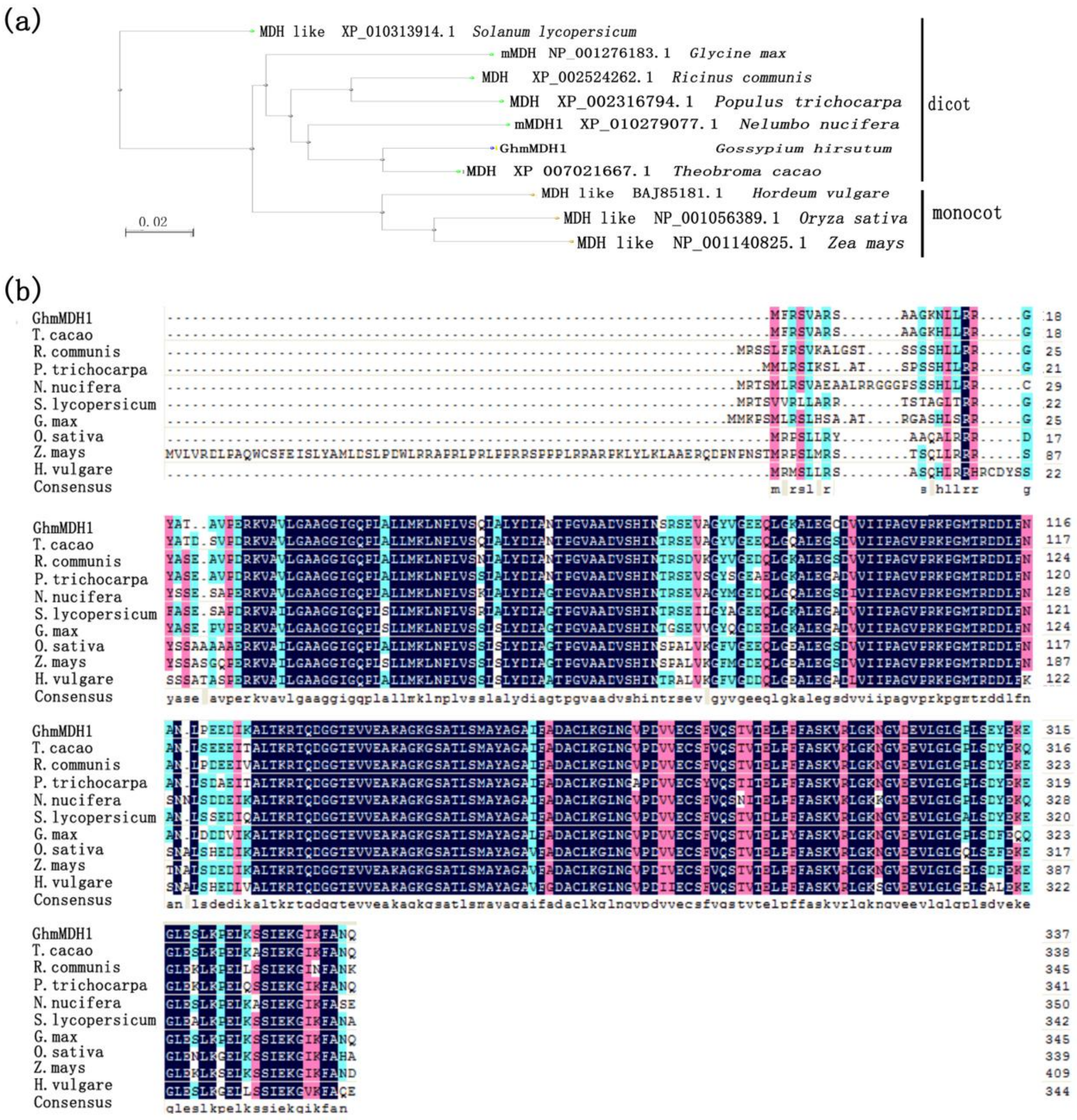
(a)
MDH like XP_010313914.1 Solanum lycopersicum
mMDH NP_001276183.1 Glycine max
MDH XP_002524262.1 Ricinus communis
MDH XP_002316794.1 Populus trichocarpa
mMDH1 XP_010279077.1 Nelumbo nucifera
GhmMDH1 Gossypium hirsutum
MDH XP 007021667.1 Theobroma cacao
MDH like BAJ85181.1 Hordeum vulgare
MDH like NP_001056389.1 Oryza sativa
MDH like NP_001140825.1 Zea mays
dicot
monocot
0.02
(b)
GhmMDH1
T. cacao
R. communis
P. trichocarpa
N. nucifera
S. lycopersicum
G. max
O. sativa
Z. mays
H. vulgare
Consensus

Figure S4.

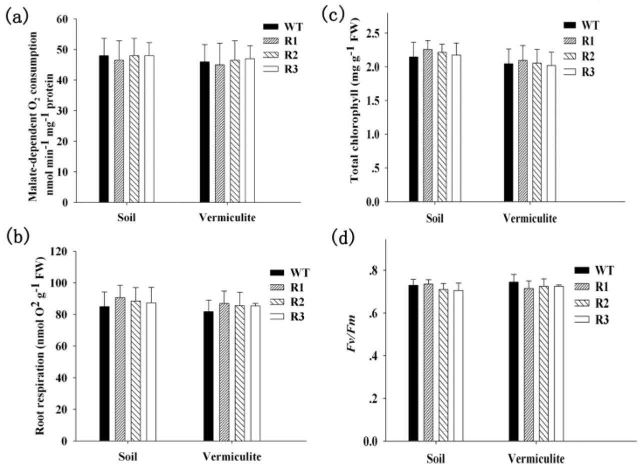

Figure S5.

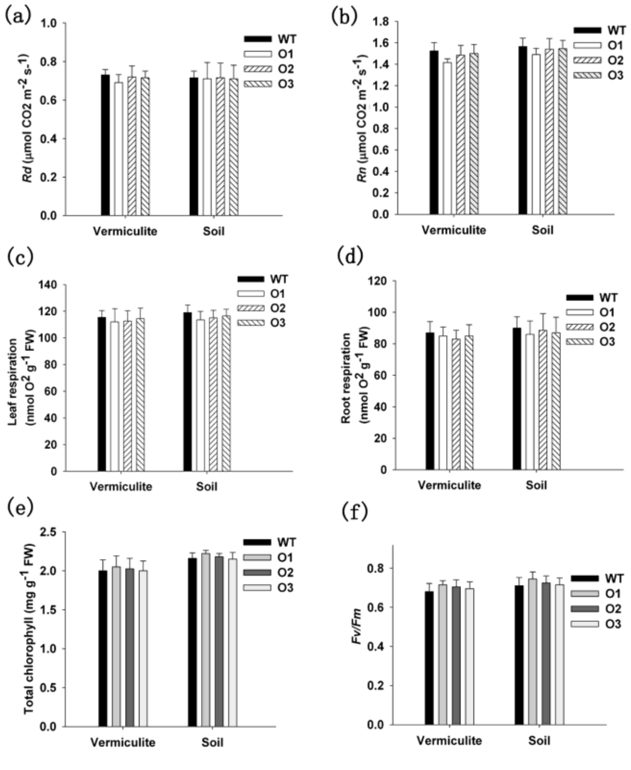

Supplement: Supplementary Information [file srep10343-s1.pdf]
